# Epithelial monolayer culture system for real‐time single‐cell analyses

**DOI:** 10.14814/phy2.12002

**Published:** 2014-04-23

**Authors:** Jong Bae Seo, Mark Moody, Duk‐Su Koh

**Affiliations:** 1 Department of Physiology and Biophysics, University of Washington, Seattle, Washington; 2 Department of Physics, POSTECH, Pohang, Republic of Korea

**Keywords:** Ca^2+^ signal, electrophysiology, epithelial culture, imaging, salt secretion

## Abstract

Many epithelial cells form polarized monolayers under in vivo and in vitro conditions. Typically, epithelial cells are cultured for differentiation on insert systems where cells are plated on a porous filter membrane. Although the cultured monolayers have been a standard system to study epithelial physiology, there are some limits: The epithelial cells growing inside the commercial inserts are not optimal to visualize directly through lenses on inverted microscopes. The cell images are optically distorted and background fluorescence is bright due to the filter membrane positioned between the cells and the lens. In addition, the cells are not easily accessible by electrodes due to the presence of tall side walls. Here, we present the design, fabrication, and practical applications of an improved system for analysis of polarized epithelial monolayers. This new system allows (1) direct imaging of cells without an interfering filter membrane, (2) electrophysiological measurements, and (3) detection of apical secretion with minimal dilution. Therefore, our culture method is optimized to study differentiated epithelial cells at the single‐cell and subcellular levels, and can be extended to other cell types with minor modifications.

## Abstract (precis)

We developed a new method to culture epithelial monolayers. This system allows high‐resolution imaging, electrophysiological measurements, and detection of secretion from differentiated epithelia.

## Introduction

Initially tissues such as dissociated frog skin were used to investigate epithelial physiology (Ussing and Zerahn 1951). Early Ussing chamber measurements of dissected epithelial monolayers vastly advanced our understanding of ion transport and salt secretion. Preparation of epithelial tissue is technically challenging and requires sacrificing multiple animals. Sample quality is paramount for data quality. These factors often result in inefficient collection and variability of data. In addition, dissected monolayers are not optimal for microscopic analysis, since structural stability is compromised in the dissection process. To circumvent these issues, investigators have developed cell lines from animal and human tissues (Simmons 1982; Widdicombe 1990; Bens and Vandewalle 2008). Using well‐established in vitro culture methods, researchers can prepare multiple monolayers of equivalent quality. Epithelial cultures grown on porous membranes develop a basolateral and apical polarization corresponding to the interstitium and lumen, respectively, of ducts of many organs. A common approach for these studies involves the use of compartmentalized culture systems which enable separate control of the apical (luminal) and basolateral (serosal) media.

The monolayer models have been useful for studying epithelial physiology for the last half century. However, for inspection of single cells with an inverted microscope, it is necessary to attempt observation of those monolayers through the filter membrane. The optical signals are degraded due to light scattering and high background fluorescence of the polymers used in the construction of the membrane. In some experiments, upright microscopes are used with the lens immersed into buffer solutions in inserts. Additionally current commercial designs (i.e., culture inserts) do not permit easy access to the cells for microscopic and electrophysiological investigation due to the plastic wall surrounding the membrane, which separates the fluid of the two compartments. The wall is tall so that even low magnification lenses with long working distance cannot focus on cells growing in the inserts.

The difficulties associated with the aforementioned model systems have resulted in heavy use of single cultured cells, especially for studying ion channels and signal transduction (Jung et al. 2004, 2009; Steward et al. 2005; Kim et al. 2008). Single‐cell cultures are typically easier, faster, and more economical to use than monolayer cultures. However, for cellular functions, differentiation is essential for replicating in vivo conditions and generating reliable data. For example, monolayers have polarized distribution of G‐protein‐coupled receptors and ion channels (Nguyen et al. 1998, 1999, 2001), and they exhibit regional Ca^2+^ signaling (Kiselyov et al. 2006; Ambudkar 2012). Therefore, it is desirable to use well‐differentiated monolayer models that incorporate physiological realities.

**Figure 1.**
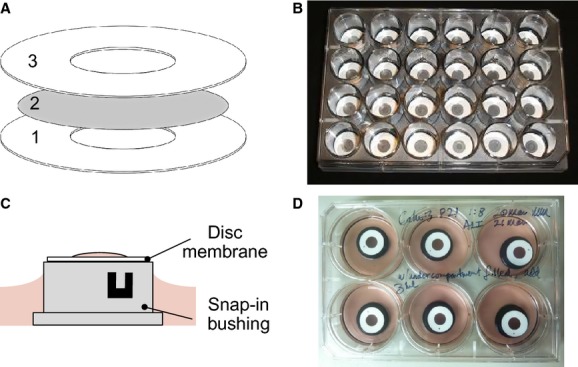
Fabrication of disk membranes and culture method. (A) Disk membrane ‘sandwich’ construction. Disks comprised three layers: two thin O‐rings with a filter membrane in the middle. See Materials and Methods for detailed information. (B) Assembled and coated disk membranes in a standard 24‐well plate ready to be used for submerged cell culture. (C, D) Air–liquid interface culture of Calu‐3 cells. A schematic diagram for a disk membrane sitting on the top of a nylon snap‐in bushing (C) and disk membranes containing confluent Calu‐3 cells in a six‐well plate (D). First, each well is filled with 4.5‐mL culture medium and a bushing (black) is added. It is necessary to take care to exclude bubbles from under the disk membrane. The disk membrane assembly allows serosal retention of approximately 1 mL of culture medium through surface tension. A portion of medium (3.5 mL) is freshly exchanged two or three times per week. Two vents on the sides of the bushing promote free exchange of medium to the basolateral surface of Calu‐3 cells.

To overcome these limitations of the current model systems, we developed a monolayer culture system which allows easy access to both sides of the epithelial monolayer. The basic idea was to lower the supporting boundary of the porous filter membrane. The goal has been achieved by adhering flat O‐rings (<100 *μ*m thick) on both sides of the filter membrane (Fig. 1). We referred to the assembled membrane as ‘disk’ membrane. We emphasize several advantages to this system: (1) The porous culture membrane is attached to a low‐profile support (<100 *μ*m) which allows easy positioning of the monolayer, including inversion. Inversion of the monolayer eliminates optical degradation in epi‐fluorescence microscopy because we can directly observe the cells without interference of the porous membrane. (2) The low profile of the support provides a small luminal volume important for analysis of secretory products without unrealistic dilution. (3) The low‐profile support and mounting system provides access for many types of probes used for physiological measurement such as transepithelial electrical resistance, patch‐clamping, and carbon fiber microamperometry. This study describes the fabrication of the disk membrane in detail, designed adaptors to microscope stage, and experimental examples using the epithelial monolayers on the disk membrane.

## Materials and Methods

### Fabrication of disk membrane

The disk membrane consists of three layers, two flat O‐rings sandwiching a filter membrane (Fig. 1A). For flat O‐rings, we use commercial adhesive reinforcement labels (Avery Dennison, 5729, Brea, CA). To begin assembly, a label is placed adhesive side up on the inside of a clean and sterile lid of a 10‐cm plastic tissue culture dish. The filter membrane (Cat. No. TMTP04700, Isopore^™^, 5 *μ*m pore size, Millipore, Billerica, MA) is cut using a 12‐mm (1/2 inch) circular punch (EK Tools, Wilton Industries, Woodridge, IL). It may be necessary to layer several membranes, including the interleaved papers that come in the filter membrane packaging, to aid in getting clean and reproducible cuts. Once the membranes are cut, grasp the edge of one membrane using fine tweezers (No. 5) for the assembly. Assembly of the disk membrane is done using a stereomicroscope as an aid for viewing. The membrane is lowered carefully to the label's adhesive surface at an angle so that one edge of the membrane contacts the surface first. Then, the membrane is carefully lowered in a slight rolling motion toward its opposite free edge, taking care to roughly center the membrane on the hole in the reinforcement label. This method is used to insure that there are no wrinkles or undulations in the membrane. We suggest using two pairs of tweezers to grasp opposing sides of the membrane and stretch the membrane flat across the adhesive of the label if necessary. Discard any imperfectly assembled membranes. Finally, center another reinforcement label carefully, adhesive side down, over the lower adhesive label/membrane assembly, taking care to match up the circular holes in the reinforcement labels. Once the label is in position, carefully apply pressure with the tweezers at several points around the label‐membrane assembly to ensure that there is good adhesion. Self‐annealing occurs slowly between adhesive layers.

The disk membrane assembly is now ready for coating. We indicated a small dot with a Sharpie permanent marker on the side to be coated for later reference. After assembly the porous portion of the disk membrane surface was coated for the attachment of cells. Most all aqueous coatings could be used. For pancreatic duct epithelial and Calu‐3 cells, the membrane was coated with PureCol (Advanced Biomatrix, San Diego, CA) (26 *μ*g/cm^2^) and human placental collagen (Bornstein and Traub Type IV; Sigma Aldrich, St. Louis, MO) (26 *μ*g/cm^2^), respectively, overnight at 4°C. The excess coating solution was aspirated and the remaining solution on the membrane allowed to dry for 30 min under UV light in a biosafety cabinet. If the underside of the disk membrane touched the tissue culture dish surface, the majority of the applied coating fluid was drawn through the membrane pores to the underside via capillary action. A cylinder such as 2–3 mm segments of a 15‐mL conical tube placed under the disk membrane assembly was used to lift the disk during the coating. The dried disk membranes could be stored before use up to 3 months at 4°C for pancreatic duct epithelial cells (PDEC).

### Cell culture

Dog PDEC were cultured as described previously (Nguyen et al. 1998). Cells were incubated with Eagle's minimum essential medium supplemented with 10% fetal bovine serum (PAA Laboratories, Dartmouth, MA), 2 mmol/L l‐glutamine, 20 mmol/L HEPES, 100 IU/mL penicillin, 100 *μ*g/mL streptomycin, 5 *μ*g/mL bovine insulin, 5 *μ*g/mL human transferrin, 5 ng/mL sodium selenite, 1% MEM vitamin solution, and 1% MEM nonessential amino acid solution (Invitrogen‐Life Technologies, Carlsbad, CA). Calu‐3 airway epithelial cells (HTB‐55) were obtained from American Type Culture Collection (ATCC) and maintained primarily according to the method in Devor et al. (1999). Cells were maintained in Dulbecco minimum essential and F‐12 media (1:1 mixture; Mediatech, Manassas, VA) supplemented with 15% fetal bovine serum, 100 IU/mL penicillin, and 100 *μ*g/mL streptomycin.

For plating cells, the coated disk membranes were placed in 24‐well cell culture plates (Cat. No. 08‐772‐1; Fisher Scientific, Pittsburgh, PA; Fig. 1B). Disk membranes were semi‐buoyant in culture medium. Therefore, for submerged culture, it was necessary to insert a sterile segment (~10 mm) of a 15‐mL conical tube to push down the disk membrane. These segments were slightly bigger than the culture wells and, therefore, had a lengthwise slit cut (~2 mm wide) into it. When inserted into the culture wells, it expanded and stayed in the well tightly.

One million cells were seeded per well. One day after plating, the disk membranes were rinsed with fresh culture medium to remove any dead cells. Calu‐3 cells were cultured until confluent (~3 days) and then placed on the protruding surface of a nylon mini snap‐in bushing, inner diameter (ID) 12.7 mm (Eagle cable mounting and accessories; Mouser Electronics, Mansfield, TX), and 6‐well culture dish (Fisher Scientific) that had been submerged in 3.5‐mL culture medium so that the cells were growing at the air–liquid interface for 14–21 days (Figs. 1C and D). Care needed to be taken to exclude bubbles from the basolateral surface of the disk membrane when placing them on the bushing. Before use, the nylon bushings and tube segments were sterilized in 70% ethanol, rinsed with distilled and deionized water, and then exposed to UV light overnight. These components could be used multiple times.

### Perfusion system and chamber

Perfusion reservoirs were constructed from 20‐mL syringes (Becton Dickinson, Franklin Lakes, NJ). Each syringe was connected to a perfusion manifold (ML‐8, Warner Instruments, Hamden, CT) using polyvinylchloride tubing (Lee Co., Essex, CT). For luminal pH measurement (Fig. 9), the entire perfusion tubing assembly was inserted into Tygon Formulation 3350 CO_2_ impermeable tubing with ID of 3.18 mm and wall thickness of 1.59 mm (Cat. No. ABW00007; Saint‐Gobain Performance Plastics, Beaverton, MI) to serve as a jacket. To ensure stable gas equilibration of perfusion fluids, the jacket was injected with 95% O_2_/5% CO_2_.

**Figure 2.**
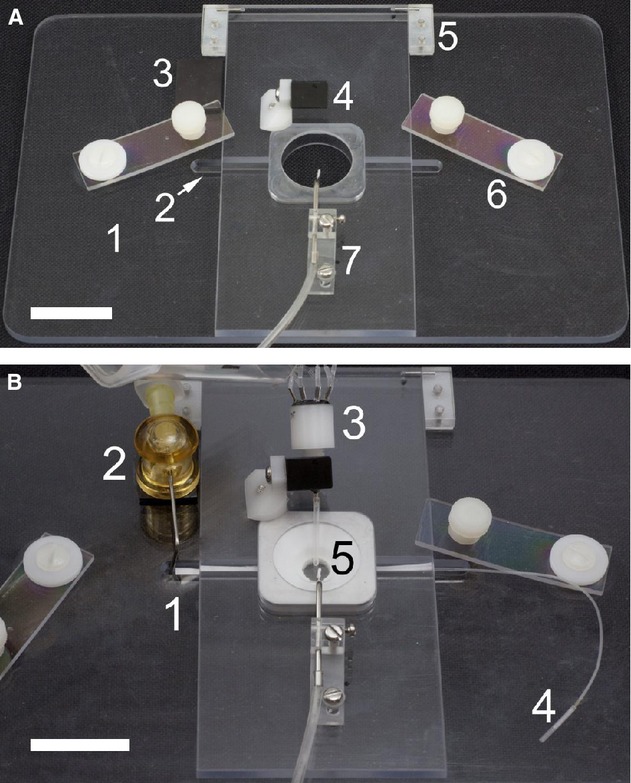
Experimental chamber for disk membranes. (A) Platform before assembly of the disk membrane. It consists of (1) stage adapter, (2) perfusion groove to accommodate inlet and outlet of lower perfusion adapter (not visible here, see Fig. 3B2), (3) magnetic tape (black) for installation of lower perfusion suction attachment (No. 2 in B), (4) adjustable perfusion manifold holder, (5) hinge to accommodate a cover to push down upper perfusion chamber, (6) tension clip for the cover (fixed to the platform by a Delrin pin on the right and a smaller plastic bolt to control push pressure on the left), and (7) adjustable suction system incorporating slit stainless steel tube. (B) Assembled perfusion system; (1) suction wick for the lower luminal chamber, (2) magnetized suction needle, (3) perfusion manifold, (4) lower perfusion inlet connector, (5) assembled unit including disk holder insert for upper perfusion, disk membrane, and lower perfusion insert (from top to bottom sequence, see Fig. 3B for its step‐by‐step assembly). Scale bar = 2 cm for both photographs.

For mounting the disk membranes on an inverted microscope, we built a custom stage mount and membrane holder (Fig. 2). For the machine shop‐generated stage mount, square # 0 coverslip (22 × 22 mm, Cat. No. 6661B52; Thomas Scientific, Swedesboro, NJ) was positioned on the holder and acted as the bottom of the chamber. Disk membrane holder inserts (Fig. 2B, component 5) were cast from a Sylgard elastomer polymer resin (3110 RTV, Dow Corning; K.R. Anderson, Morgan Hill, CA) using a mold that was milled by a fine mechanical machine shop. For the suction in the upper chamber, a round filter paper (5 mm, e.g., osmometer sample disks, Wescor, Logan, UT) was used since an open needle tip generates intermittent aspiration of buffer in the chamber. This wick positioned on the slope of the chamber promoted smooth continuous suction.

**Figure 3.**
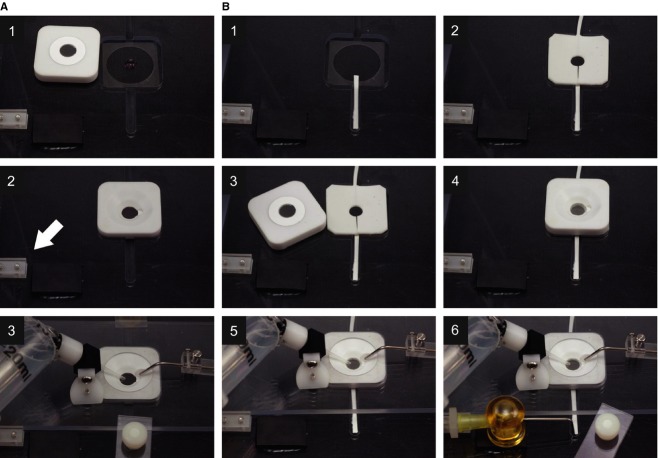
Assembly of disk membrane and chambers. Sequential images for single (A)‐ and double (B)‐sided perfusion experiments. (A) Perfusion from the upper side of the monolayer. Step 1: Place a standard coverslip (#0 or #1) over the opening of stage adaptor. Load a drop of luminal or serosal solution (10–20 *μ*L) at the center of the coverslip. Remove a disk membrane from culture and gently rinse in 5 mL of the luminal solution. Place the disk membrane on the white upper chamber with cells oriented either up or down as suits your experimental purpose. The disk membrane, due to surface tension of the buffer, will adhere to the upper chamber as shown. However, it may be helpful to use a Kimwipe or paper towel to wick away extra fluid from the disk membrane ring before placing on the upper chamber surface. Step 2: Place upper chamber and disk membrane on the stage adaptor. Step 3: Gently lower the upper part of the clamshell chamber (acrylic push cover) into position and secure the tension clip. Note that inlet and outlet needles are preadjusted and positioned to the same positions when the push cover is locked. Insert a round filter paper between suction needle and a notch for smooth suction of bath solution. (B) For double‐sided perfusion, a Sylgard lower chamber needs to be installed underneath the disk membrane. Step 1: Place a wick which will allow pressure‐free removal of the lower chamber perfusate. Step 2: Apply vacuum grease, sparingly to avoid squeeze‐out, to the underside of the lower chamber and gently press it on the coverslip so that the split in the lower chamber overlaps the wick, allowing for perfusate exit. Step 3: Open the valve to the lower chamber perfusate and allow the reservoir to be slightly over filled with the luminal or serosal solution. At this point the wick should not be in contact with the perfusate so that surface tension creates a small dome on the perfusate in the lower chamber. Place the disk membrane on the upper chamber. Step 4: Place the upper chamber/disk membrane onto the lower chamber, one side first, to avoid bubbles in the lower chamber. Step 5: Close the clamshell chamber. Step 6: After positioning the lower chamber suction (yellow needle) just beyond the end of the wick and securing the tension clips, begin perfusion with control solutions.

Figure 3A provides a step‐by‐step procedure to install a disk membrane for upper chamber perfusion only. To perfuse the lower side of the monolayer, we constructed an additional luminal perfusion adapter (Fig. 3B2). The adapter was made of the same Sylgard resin. The adapter was cast on a flat plastic or glass surface using a stack of four #0 coverslips as spacers between the surface and a glass slide. A segment of PE 10 tubing about 6 cm long (Becton Dickinson, Sparks, MD) was inserted between the surface and the plate with both sides sticking out of the casting chamber. After injection of resin, polymerization, and punching a 6 mm hole with a circular punch, the PE 10 tubing on one side of the hole was cut out of the adapter with a razor blade, leaving a slit for collection of perfusate by leakage. Then, rectangular borders were trimmed with the blade. A cut strip of Whatman #1 qualitative filter paper (visible in Fig. 3B1) was used to remove leaking solution from the lower compartment. We estimated solution exchange measured with the clearance of trypan blue. Time required for 20–80% solution exchange was 11.5 s at the flow rate of 160 *μ*L/min at the upper chamber and 19.5 s at 100 *μ*L/min for the lower chamber. Therefore, test solutions could be applied within 30–40 s.

### Measurement of transepithelial electrical resistance (TER)

TER of epithelial monolayer grown on disk membrane was measured with EVOM^™^ epithelial voltohmmeter (World Precision Instruments Inc., New Haven, CT) and recorded with the analog‐digital board of an EPC9 patch‐clamp amplifier (HEKA Elektronik, Lambrecht, Germany). The amplifier used two pairs of Ag:AgCl electrodes printed on thin plastic strip (‘chopstick electrodes’). The first one was positioned below the lower chamber (Fig. 3B2) and sandwiched by an additional flat pad similar to the lower chamber without the center hole. The assembly was sealed with grease to block leakage of solution. The other electrode was positioned in the upper chamber using a manipulator. Background resistance of solutions and a disk membrane without cells were subtracted and TERs are expressed as ohm times area of monolayer (Ω·cm^2^). For example, TER of our intact PDEC monolayers was 344 ± 21 Ω·cm^2^ (*n* = 2). The number decreased after permeabilization of the basolateral membrane (181 ± 14.7, *n* = 4, Fig. 6A). When cultured on commercial inserts, TER value of intact PDEC monolayers was reported to be around 700 Ω·cm^2^ (Okolo et al. 2002), twice larger than that of the monolayers grown on our disk membrane. This difference may be due to different thicknesses of collagen, a major component of the extracellular matrix for differentiation, proliferation, and attachment of cells. Typically a thick (>1 mm) and polymerized Vitrogen layer is used for the culture in Transwell inserts. For the disk membrane, we applied less Vitrogen and air dried to maintain the coating of <0.09 mm.

Calu‐3 monolayer is cultured in two different ways; liquid‐covered culture (LCC) and air‐interfaced culture (AIC). AIC is supposed to be a representative model of the airway epithelium (Yamaya et al. 1992; Johnson et al. 1993; Sachs et al. 2003; Widdicombe et al. 2003; Grainger et al. 2006). Grainger et al. (2006) measured the change of TER systematically. The maximal TER of Calu‐3 monolayer was 1086 ± 113 Ω·cm^2^ for LCC, while the value decreases by three times (306 ± 53 Ω·cm^2^) when grown in AIC condition for 11–13 days. Since our Calu‐3 monolayers were maintained in AIC for 3 weeks, and further permeabilized from one side, the initial TER of our monolayer (225 ± 23 Ω·cm^2^, Fig. 6A) seems to be comparable to the reported value. We also confirmed that the contact between the adhesive and filter membrane is electrically tight (3389 ± 11 Ω·cm^2^, *n *= 4) using the disk membranes made of a thin plastic layer (without holes) instead of the filter membrane. All quantitative values in TER and other experiments are expressed as mean ± standard error of means.

### Ca^2+^ imaging

Intracellular Ca^2+^ level was measured with Fluo‐4 Ca^2+^‐sensitive dyes (Invitrogen‐Life Technologies). Dog PDEC monolayers grown for 3 days on the disk membranes and PDEC single cells for 1 day on glass chips were loaded with 8 and 2 *μ*mol/L Fluo‐4 acetomethoxy (AM), respectively, containing 0.2% pluronic acid for 30 min at room temperature. Cells were then allowed an additional de‐esterification incubation period of 30 min before recording. Fluo‐4‐loaded cells were excited with 488‐nm light and emission was collected at >492 nm using a confocal microscope (LSM 710, Carl Zeiss, Oberkochen, Germany) and analyzed with Igor Pro (WaveMetrics, Portland, OR).

### Measurement of cAMP

Calu‐3 cells were dissociated with 0.25% Trypsin‐EDTA, divided (2 × 10^6^ cells/tube), and incubated with or without 10 *μ*mol/L forskolin for 20 min. Intracellular cAMP level was estimated using a competitive immunoassay kit (Cat. No. KGE002B; R&D Systems, Minneapolis, MN) according to the manufacturer's instruction.

### Measurement of luminal pH

Luminal pH was measured as described previously (Ishiguro et al. 1996) with minor modifications. Briefly, 10 *μ*L of freshly prepared luminal buffer including 50 *μ*mol/L 2′,7′‐bis‐(2‐carboxyethyl)‐5‐(and‐6)‐carboxyfluorescein (BCECF)‐dextran (70,000 MW; Invitrogen‐Life Technologies) was placed on glass bottom. High‐molecular‐weight BCECF was used to minimize its diffusion or transport through the plasma membrane. BCECF‐dextran was excited at 440 and 495 nm and fluorescence emission was collected at 535 ± 20 nm in an epifluorescence system with an inverted microscope (TE2000‐U, Nikon, Melville, NY) attached to an Evolve 512 EMCCD (Photometrics, Tucson, AZ). The ratio of fluorescent emissions with 440‐ and 495‐nm excitations was linear between pH 6 and 8, the physiologically relevant range.

The buffers used for luminal pH studies were designed to optimize potential bicarbonate secretion into the space on the side of the monolayer corresponding to the duct lumen. Luminal buffer included (in mmol/L): 140 NaCl, 5 KCl, 1.2 MgCl_2_, 1.2 CaCl_2_, 1 HEPES, 10 glucose, 9 mannitol, pH 7.2. The luminal buffer, which contained no bicarbonate and was weakly buffered with only 1 mmol/L HEPES, was designed to be sensitive to pH changes and therefore it was prepared freshly before use. The pH was checked and retitrated, if necessary. Serosal bicarbonate buffer had 120 NaCl, 5 KCl, 1.2 MgCl_2_, 1.2 CaCl_2_, 25 Na_2_HCO_3_, 10 glucose, pH 7.4 at 23°C when bubbled with 95% O_2_/5% CO_2_. Serosal bicarbonate‐free buffers contained 140 NaCl, 5 KCl, 1.2 MgCl_2_, 1.2 CaCl_2_, 10 HEPES, 10 glucose, pH 7.4.

### Carbon fiber microamperometry

To detect exocytosis from PDEC, cells were loaded with 70 mmol/L dopamine in Ringer's solution for 40 min at room temperature (Koh et al. 2000). The molecules are accumulated in acidic secretory vesicles and released upon exocytosis of the vesicles. To adjust osmolarity of the loading solution, equimolar NaCl was replaced by 70 mmol/L dopamine (pH 7.3). To minimize spontaneous oxidation of dopamine, 2 mmol/L ascorbic acid was included in the loading solution. Cells were washed with dopamine‐free Ringer's and used within 2 h after dopamine loading. Miniature carbon fiber electrodes with a 11 *μ*m tip diameter were fabricated as described previously (Koh 2006). Electrode potential was set as +400 mV to oxidize dopamine. Amperometric current was recorded using with an EPC9 patch‐clamp amplifier with filter and sampling frequencies of 100 and 500 Hz, respectively.

### Chemicals and solutions

Chemicals were purchased from Sigma‐Aldrich unless otherwise specified. Standard Ringer's saline for all experiments other than luminal pH measurement was composed of (in mmol/L) 137.5 NaCl, 2.5 KCl, 1 MgCl_2_, 2 CaCl_2_, 10 HEPES, 10 glucose, pH 7.3.

## Results

**Figure 4.**
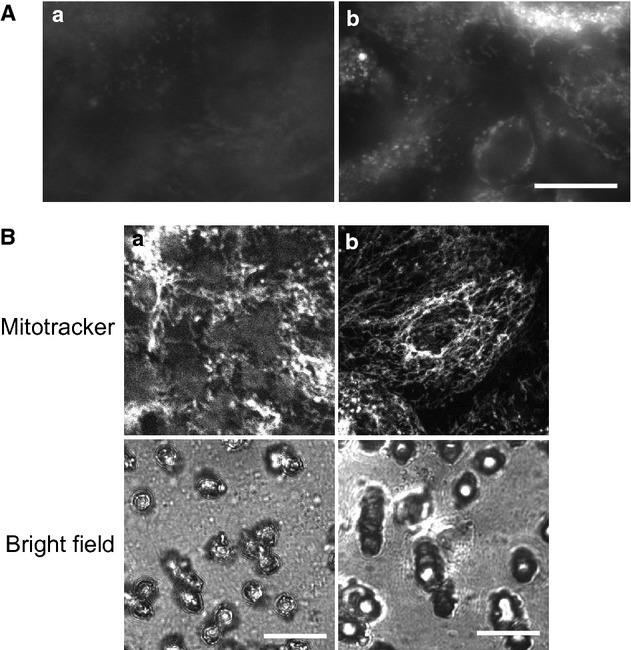
Comparison of fluorescence images with and without interfering plastic membrane. (A) Epifluorescence micrographs of PDEC monolayers. After labeling with 2 *μ*mol/L FM 1‐43 (Cat. No. F‐35355, Invitrogen‐Life Technologies) for 6 h in culture medium, PDEC monolayers were illuminated and visualized through the supporting filter membrane of the insert (a) and directly without the filter membrane (b). Images were obtained with a 20× oil lens (Plan Fluor, NA 0.75, Nikon) and an inverted epifluorescence microscope (TE2000‐U, Nikon) equipped with Evolve 512 EMCCD (Photometrics). (B) Confocal micrographs of PDEC monolayers. PDEC monolayers were labeled with 50 nmol/L Mitotracker Red CMXRos (Cat. No. M‐7512, Invitrogen‐Life Technologies) for 15 min in culture medium at 37°C and then obtained with a 63× oil lens (Plan Apochromat, NA1.40) and Zeiss 710 laser‐scanning confocal microscope. Scale bar = 20 *μ*m

We performed several experiments to test the functionality of our disk membrane culture system. First, we compared fluorescent images of PDEC monolayers observed directly or through supporting filter membrane with epifluorescence (Fig. 4A) and confocal fluorescence microscopies (Fig. 4B). To label intracellular organelles, cells were labeled with FM 1‐43 fluorescent membrane dye (Fig. 4A) or Mitotracker Red CMXRos (Fig. 4B). The FM 1‐43 dye incorporated into the plasma membrane had been recycled by endocytosis and labeled several intracellular organelles including secretory vesicles (Taraska and Almers 2004; Jung et al. 2009). The specific monolayer shown in Figure 4Aa was grown in the commercial 12 mm Snapwell insert (Cat. No. 3407; Corning Costar, New York, NY) and, therefore, it could be visualized only through the supporting filter membrane. The image was not clear due to diffraction and light scattering from the opaque membrane and pores. In addition, background fluorescence was too high to obtain a high contrast fluorescent image. By comparison, epithelial monolayers visualized directly without the filter membrane revealed intracellular organelles, round structures (secretory granules), and tubular structures (endosome or endoplasmic reticulum; Fig. 4Ab). This high‐resolution image could be easily obtained since our disk membrane culture allowed us to invert the membrane and focus on the cells in monolayers using an inverted microscope. The resolution of fluorescence image was further increased when the monolayers were observed under a confocal microscope (Fig. 4B). PDEC monolayer was stained with Mitotracker Red CMXRos to label mitochondria. The confocal image visualized directly without interference from the filter membrane clearly revealed the worm‐like structure of mitochondria (Fig. 4Bb). However, when observed through the filter membrane, the image was seriously distorted, especially near the pores of the filter membrane (Fig. 4Ba). In addition, the direct observation of the monolayer included less background fluorescence from the filter membrane and therefore provided a better contrast.

**Figure 5.**
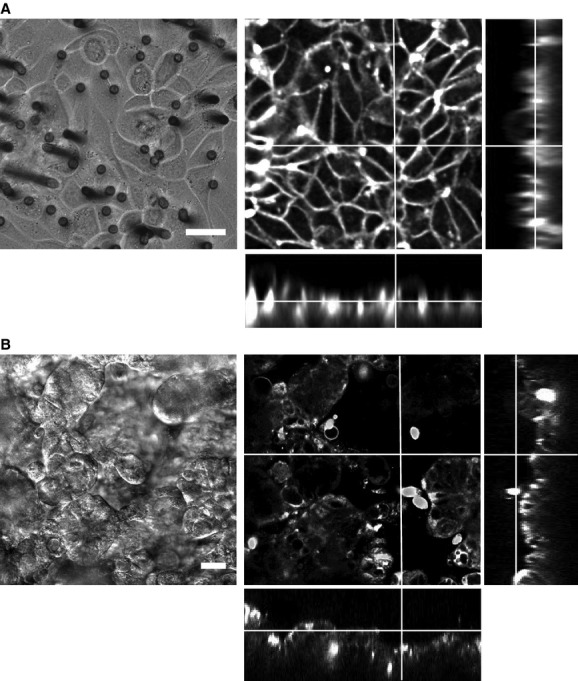
Confocal fluorescence microscopy of epithelial monolayers. Polarized PDEC (A) and Calu‐3 (B) monolayers. Left micrographs are confocal differential interface contrast images with the luminal membrane facing down. Right micrographs are confocal images with z‐stack profiles for x‐ and y‐axes. The monolayers were stained with 8 *μ*mol/L FM 1‐43 in the luminal buffer for 30 min. FM 1‐43 dye was excited at 510 nm and fluorescence emission was detected at 626 nm. Fluorescence images were improved by digital deconvolution using Image J software. Scale bar = 20 *μ*m.

Next, to check the morphology of epithelial monolayer, a PDEC monolayer on the disk membrane was flipped over so that luminal side was pointing down toward the glass bottom of the chamber. The plasma membrane was quickly stained with FM 1‐43 dye included in the luminal buffer and became fluorescent. Confocal Z‐sectioning and a reconstructed image demonstrated that PDEC form a relatively flat layer with cells overlapping each other to some extent (Fig. 5A). In contrast, with Calu‐3, an airway serous cell line, some areas were devoid of cells at the specific focal plane (Fig. 5B). The Z‐sectioning revealed that the cells formed corrugated monolayers after 2 weeks of culture in the air–liquid interface (Materials and Methods and Fig. 1C and D). This Calu‐3 monolayer surface was typical of those seen by others (Sondergaard et al. 2008; Stentebjerg‐Andersen et al. 2011).

**Figure 6.**
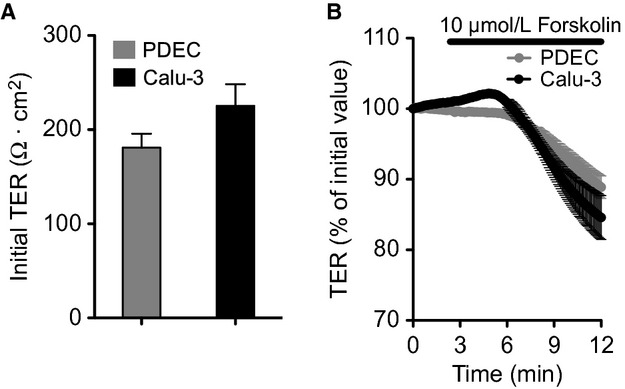
Measurement of transepithelial electrical resistance (TER) of PDEC and Calu‐3 monolayers after basolateral permeabilization with Amphotericin B (0.5 mg/mL). (A) Initial TER of PDEC and Calu‐3 monolayer. (B) TER of both monolayers was reduced by 10 *μ*mol/L forskolin (*n* = 4 monolayers for each cell type).

To test whether our disk membrane culture and chamber systems can determine the change of electrogenic transport through the membrane of epithelial monolayer as a conventional Ussing chamber experiment, we measured transepithelial electrical resistance (TER) after the permeabilization of the basolateral membrane with Amphotericin B. The initial TER of PDEC and Calu‐3 monolayer were 181 ± 14.7 and 225 ± 22.9 Ω·cm^2^, respectively (Fig. 6A). The TER was reduced upon forskolin application consistent with previous reports (Fig. 6B; Devor et al. 1999; Nguyen et al. 2001). The reduction of TER by forskolin may be due to either the increase of the conductance of cAMP‐activated channels such as CFTR or an increase in paracellular permeability by cAMP.

**Figure 7.**
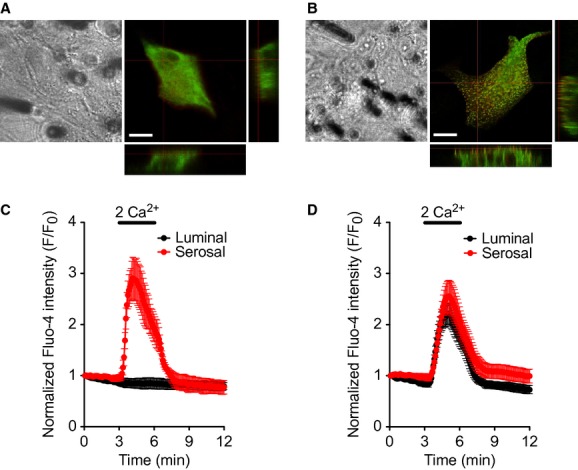
Monitoring of SOC proteins in PDEC monolayers. (A, B) Confocal images of PDEC transfected with Orai1‐Orange and STIM1‐GFP. Control (A) and after depletion of Ca^2+^ stores with 5 *μ*mol/L thapsigargin for 15 min in Ca^2+^‐free Ringer's solution (B). X–Z and Y–Z reconstructions were obtained across the nucleus voids to better separate the apical and basolateral membranes. A transfected cell surrounded by untransfected cells was chosen for both conditions. Scale bar = 20 *μ*m. (C, D) Confocal Ca^2+^ imaging of PDEC monolayers loaded with Fluo‐4 Ca^2+^ dye to localize the functional SOC. Untransfected (C) and Orai1‐Orange and STIM1‐GFP‐transfected (D) monolayers were treated with thapsigargin for store depletion for SOC activation and then perfused with Ringer's without Ca^2+^ (with 1 mmol/L EGTA) and subsequently with Ringer's containing 2 mmol/L Ca^2+^.

With the high resolution of confocal microscopy, it was possible to study the localization of specific proteins inside single cells in a monolayer (Fig. 7). In this imaging experiment, we monitored cellular locations of overexpressed Orai1 and STIM1 proteins for store‐operated Ca^2+^ channels (SOC) in PDEC (Feske et al. 2006; Cahalan et al. 2007). The proteins were tagged with Orange and green fluorescence protein (GFP), respectively. Before store depletion, control cells showed Orai1‐Orange channels mainly in the plasma membrane and STIM1‐GFP Ca^2+^ sensors inside the cell (probably in the endoplasmic reticulum, the major Ca^2+^ store in PDEC; Fig. 7A). After treatment of PDEC monolayers with 5 *μ*mol/L thapsigargin, a blocker of sarco‐/endoplasmic reuptake Ca^2+^ pump, in Ca^2+^‐free Ringer's solution to deplete Ca^2+^ stores, two proteins formed puncta as typically observed for the activation of SOC (Cahalan 2009; Fig. 7B). Z‐scan images suggested an apical localization of Orai1‐STIM1 puncta. This result was not expected based on our previous finding that activated SOC are localized in the basolateral membrane (Kim et al. 2013). We interpreted that overexpression of Orai1 proteins, typically 10–100 times more than endogenous proteins, results in mislocalization of the protein to the apical side. To test the hypothesis, we examined on which side the functional SOC are formed before and after overexpression. First, SOC were activated by 5‐min treatment with thapsigargin and Ca^2+^ applied to either apical or basolateral side of the monolayer. With control untransfected monolayers, Ca^2+^ influx through SOC and, therefore, intracellular Ca^2+^ increase was observed only with serosal Ca^2+^ application (Fig. 7C). This location is consistent with basolateral endogenous SOC puncta visualized with an Orai3‐specific antibody (Kim et al. 2013). By contrast, in the monolayers with Orai1‐STIM1 overexpression, both apical and basolateral Ca^2+^ raised intracellular Ca^2+^ levels, implicating that apically localized exogenous Orai1 forms functional SOC at the apical membrane, along with basolateral endogenous SOC proteins (Fig. 7D).

**Figure 8.**
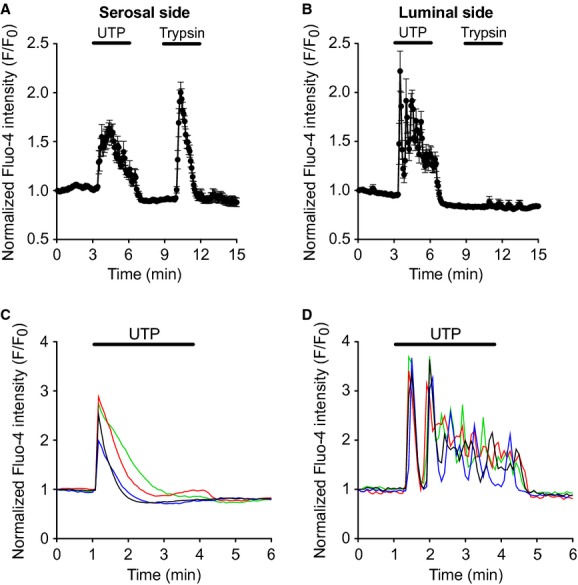
Measurement of intracellular Ca^2+^ signals of PDEC. Confocal Ca^2+^ measurements using Fluo‐4 dye for monolayers and single cells. Relative Fluo‐4 intensity was calibrated to the initial fluorescence levels of each cell. (A, B) Confocal Ca^2+^ imaging of PDEC monolayers upon activation of serosal and luminal purinergic and PAR‐2 receptors using 100 *μ*mol/L UTP and 1 *μ*mol/L trypsin, respectively (*n* = 3 monolayers and 6–7 single cells per monolayer). Ca^2+^ response of a single isolated cell (C) and neighboring single cells in a monolayer (D) upon 100 *μ*mol/L UTP. For the monolayer experiment, UTP was applied to the luminal side. The same experiment is shown in Movie S2.

Our culture system is optimal for measuring intracellular signals in individual cells of an epithelial monolayer. Intracellular Ca^2+^ signaling has been well studied in PDEC mostly using dissociated cells (Jung et al. 2004, 2009; Kim et al. 2008). Although single‐cell Ca^2+^ signaling and dynamics are fairly well known, basic understanding about Ca^2+^ signaling in well‐differentiated monolayers is limited at the cellular level and questions about organ level function or dysfunction remain unexplored (Ambudkar 2012). Here, as a test of our system, we measured Ca^2+^ signals in PDEC monolayers generated by stimulation of apical (luminal) and basolateral (serosal) receptors. PDEC cells were loaded with Fluo‐4 Ca^2+^ dye. Increase of fluorescence intensity at 488‐nm excitation wavelengths indicates increase in intracellular Ca^2+^ concentration ([Ca^2+^]_i_). PDEC express P2Y_2_ purinergic receptors coupled to G_q_ and phospholipase C (PLC). PLC, in turn, cleaves PIP_2_ to generate IP_3_ and diacylglycerol. IP_3_, after binding to IP_3_ receptors in intracellular Ca^2+^ stores, mobilizes Ca^2+^ to the cytoplasm as we have analyzed in previous studies (Jung et al. 2004). The purinergic receptors are in both apical and basolateral membranes of PDEC (Nguyen et al. 1998, 2001). Protease‐activated receptor 2 (PAR‐2) is also PLC coupled and raises [Ca^2+^]_i_ (Nguyen et al. 1999; Kim et al. 2008). However, PAR‐2 is located only on the basolateral side. We confirmed the previous findings using the monolayers on disk membranes. By activating purinergic receptors, UTP could evoke Ca^2+^ signals from both sides while trypsin acting on PAR‐2 was efficient only from the serosal (basolateral) side (Fig. 8A and B, Movies S1, S2). In undifferentiated and isolated single PDEC, a high concentration of UTP such as 100 *μ*mol/L evoked a peak Ca^2+^ response followed by a plateau phase (Fig. 8C). By contrast, the same concentration of UTP evoked oscillatory Ca^2+^ signals in PDEC in a monolayer (Fig. 8D, Movie S2), suggesting Ca^2+^ signaling in monolayers is not identical to that in dissociated cells. Interestingly, the Ca^2+^ signals in neighboring cells were synchronized perhaps due to gap junctional connection in PDEC (Oda et al. 1996) as observed in many different types of tissues (Leite et al. 2002; Ravier et al. 2005; Fig. 8D and Movies S1, S2).

We also could measure intracellular pH in PDEC in monolayers after loading with BCECF‐AM dyes (data not shown), as we did with single PDEC (Jung et al. 2006). In sum, intracellular signals in epithelial monolayers can be easily monitored in real‐time at single‐cell resolution.

**Figure 9.**
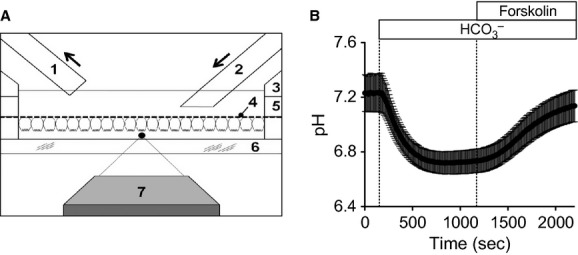
Measurement of luminal pH of Calu‐3 epithelial monolayers using BCECF‐dextran. (A) A schematic diagram of assembled chamber including cells mounted and oriented to form an ‘artificial duct’. Not drawn to scale. In this example, the system is configured only for serosal (basolateral) perfusion of the membrane with (1) suction, (2) perfusion inlet, (3) disk membrane holder insert (i.e., upper chamber), (4) porous membrane with attached cells, (5) white outer rim portion of disk membrane (the same component as 1 and 3 from Fig. 1A), (6) glass coverslip, and (7) microscope objective. Black dot indicates approximate focal plane for BCECF experiments. (B) pH of luminal buffer adjacent to the apical membrane of Calu‐3 cells. Initial perfusion of a serosal bicarbonate‐free buffer was followed by a serosal bicarbonate buffer gassed with 95% O_2_/5% CO_2_, and then followed by the addition of 10 *μ*mol/L forskolin (*n* = 7 monolayers).

Next, we tested whether our disk culture method is suitable for the measurement of secretion from epithelial cells. The rationale for this assay was that secreted products from epithelial cells would not be significantly diluted in the small luminal volume of our disk membrane (about 3 *μ*L) and therefore detectable by reporter molecules. In this example, we tried to estimate apical HCO_3_^−^ (or H^+^) secretion from Calu‐3 cells (Illek et al. 1997), a phenomenon which is well characterized and is similar to distal pancreatic duct epithelia (Steward et al. 2005). Unlike many previous studies using intracellular BCECF to calculate HCO_3_^−^ secretion, we directly measured luminal pH. This method was originally used by Ishiguro et al. (1996) to measure luminal HCO_3_^−^ secretion by isolated pancreatic ducts. In our experiment, a dextran‐conjugated form of BCECF in the luminal buffer reported pH change due to fluid secretion including HCO_3_^−^ and H^+^ ions from a Calu‐3 monolayer (Fig. 9). Initially, the luminal pH was around 7.2 when the serosal side was perfused with serosal bicarbonate‐free buffer (Fig. 9B). Switch to a serosal bicarbonate buffer acidified the luminal buffer since excessive CO_2_ in the serosal buffer crosses the monolayer quickly and generates HCO_3_^−^ and H^+^. The pH recovery was minimal during continuous perfusion of serosal buffer containing bicarbonate. If Calu‐3 cells secrete HCO_3_^−^ apically using their transporters and intracellular carbonic anhydrases or reduce H^+^ efflux into the luminal compartment, we expect an increase in luminal pH. Indeed, subsequent perfusion of bicarbonate buffer with forskolin, an adenylyl cyclase activator, accelerated the pH recovery. The same concentration of forskolin increased cAMP level in Calu‐3 cells as measured with an ELISA kit (21.2 ± 1.8 pmol/10^6^ cells in control and 683.8 ± 4.4 pmol/10^6^ cells in forskolin‐treated cells, *n* = 3). The luminal alkalinization was not repeated by 1,9‐dideoxyforskolin, an inactive analog of forskolin (data not shown). These results suggest that the forskolin‐induced luminal alkalinization is cAMP dependent. The luminal alkalinization can be mediated by upregulation of cystic fibrosis transmembrane conductance regulator (CFTR; Illek et al. 1997; Inglis et al. 2002; Ishiguro et al. 2009) or downregulation of sodium–hydrogen exchanger (NHE) by cAMP (Dudeja et al. 1999; Al‐Bazzaz et al. 2001; Inglis et al. 2002). In our preliminary results, the alkalinization was not blocked by CFTR inhibitors (CFTRinh‐172 and glibencamide) but was reduced significantly by an NHE inhibitor (5‐(N‐ethyl‐N‐isopropyl)‐amiloride), suggesting that NHE contributes to the luminal pH change. This possible involvement of NHE is supported by the previous findings that NHE is inhibited by cAMP (Pollock et al. 1986; Cano et al. 1993; Zhao et al. 1999) and NHE1 is expressed in human airways (Dudeja et al. 1999). Apical NHE activity also was suggested in several respiratory epithelial cells (Sano et al. 1988; Shaw et al. 1990; Acevedo and Steele 1993; Oelberg et al. 1993; Urbach et al. 2002) *cf*. basolateral localization in (Smith and Welsh 1992). The exact location of NHE in Calu‐3 monolayer is not determined yet. Alternatively cAMP increased paracellular permeability and thereby elevated luminal pH (Perez et al. 1997; Weiser et al. 2011). In sum, the underlying mechanisms for the luminal alkalinization need further investigation in depth.

**Figure 10.**
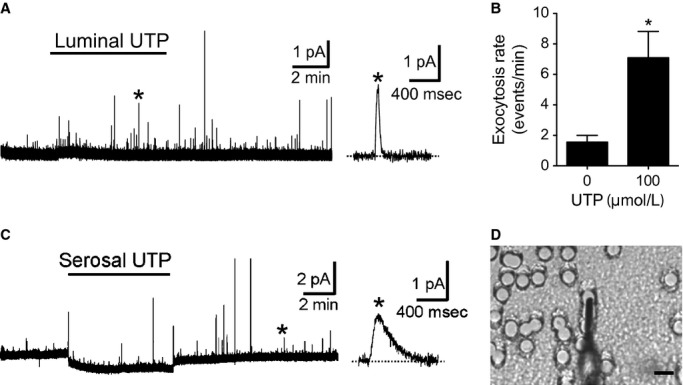
Microamperometry to detect exocytosis from differentiated PDEC. (A) Apical (luminal) exocytosis was triggered by luminal application of 100 *μ*mol/L UTP and measured with a carbon fiber microelectrode. (B) Apical exocytosis was analyzed before and during UTP application (*n* = 6 monolayers). (C) Basolateral exocytosis was evoked by serosal application of 100 *μ*mol/L UTP. Single amperometric events are shown with an expanded timescale on the right. (D) For basolateral access, a carbon fiber microelectrode was placed through the pores of the filter membrane. Scale bar = 20 *μ*m.

Finally, we confirmed that our monolayer system is compatible with the use of electrodes. Figure 10 illustrates amperometric records from PDEC monolayers. Cells were preloaded with oxidizable dopamine, a false reporter, to detect exocytosis from PDEC (Koh 2006). Apical exocytosis was detected when a miniature carbon fiber electrode (CFE) approached from the luminal side (Fig. 10A and B). In control condition, there were amperometric spikes at a low frequency, indicating that basal exocytosis is rare (1.6 ± 0.4 events/min). When cells were stimulated with luminal application of UTP, more exocytotic events were detected (8.1 ± 1.7 events/min). These single vesicle fusion events were relatively fast in rising and decay times as observed with single cells (Jung et al. 2004, 2010). When a disk membrane was installed with serosal side up, we could measure the basolateral exocytosis. To touch the basolateral side, we cultured the monolayer on a filter membrane with a bigger pore size (10 *μ*m instead of commonly used 3–5 *μ*m). These big pores, especially the ones connected in a row, allowed CFE to penetrate the filter membrane and approach the basolateral side of PDEC (Fig. 10D). Here, again we see an increase in exocytosis upon serosal UTP stimulation (Fig. 10C). However, individual amperometric spikes were slow as if the release site was remote from the tip of the CFE, presumably due to the thick collagen layer between the cells and the filter membrane. Alternatively, these events may have originated from the lateral sides of PDEC and been diffused before reaching the CFE that was touching the basal membrane.

In summary, our results highlight the advantages of our novel epithelial culture system: single cell imaging with a high spatial resolution, detection of secretory products, and use of miniature electrodes.

## Discussion

We described the manufacture of newly designed disk membrane and experimental chamber system. Although we have examined two different epithelial cell types, PDEC and Calu‐3, the same culture method can potentially be used for all epithelial cells and other types of cells capable of growing on filter membranes.

### Fabrication of disk membranes and chambers

With some exercise, the disk culture membranes are easily prepared partly due to the use of commercial reinforcement labels. The disk membranes are also cost effective ($0.2/each). One side of the label is already coated with an even adhesive layer. This glue layer is nontoxic to the cells and remains tight over a few weeks of culture so that both sides of a monolayer can be separately perfused without solutions being mixed. The disk membrane consists of two layers of the labels and is mechanically stable enough to be handled during cell culture and installation into the measuring chambers. After sterilization with UV light, the membranes could be used for long‐term culture (longer than a few weeks) when a standard concentration of antibiotics is included in the culture medium. The chambers constructed with Sylgard elastomer were used to install the disk membrane and to perfuse test solutions to both sides of epithelial monolayers during continuous real‐time measurements. With some degree of polymer flexibility, these chamber blocks act as gaskets for tight sealing as well.

### Imaging and electrochemical measurements of epithelial monolayers on the disk membranes

We presented some examples to demonstrate that our culture method on disk membrane is useful for both optical and electrochemical measurements. These results revealed that cellular morphology and protein localization (Figs. 5, 7), membrane permeability (Fig. 6), intracellular signals (Fig. 8), fluid secretion (Fig. 9), and exocytosis (Fig. 10) from differentiated individual cells can be studied with high spatiotemporal resolution. These measurements are not possible or challenging with the monolayers prepared on commercial inserts. For example, use of less opaque polyester membrane in the inserts improved the visibility of monolayers through the membrane but does not remove the deformation of images around pores or background fluorescence coming from the plastic material.

Using dissociated single cells, we and others have dissected Ca^2+^ signaling pathways in PDEC and identified several G‐protein‐coupled receptors coupled to G_q_ proteins and phospholipase C, including P2Y_2_ purinergic (Jung et al. 2004, 2009, 2010; Steward et al. 2005) and PAR‐2 receptors (Nguyen et al. 1999; Kim et al. 2008). After polarization, PDEC express the same set of receptors but new aspects such as their localization (e.g., apical vs. basolateral) arise (see Fig. 8). [Ca^2+^]_i_ oscillates in differentiated monolayers, probably due to cell‐to‐cell connections by gap junction or autocrine/paracrine signaling. Understanding the oscillatory nature of Ca^2+^ signaling and how that information is translated to Ca^2+^‐dependent mechanisms is important (Parekh 2011). In addition, a comparison of [Ca^2+^]_i_ in nearby and adjacent individual cells revealed a certain level of intercellular synchronization of Ca^2+^ signaling. Development of a new monolayer culture and chamber system allows study of Ca^2+^ signaling to proceed to the next level of complexity. The different profiles of Ca^2+^ in response to 100 *μ*mol/L UTP in single and differentiated PDEC (Fig. 8C and D) and establishment of serosal PAR‐2 location (Fig. 8A and B) indicate the importance of studying Ca^2+^ signaling in well‐differentiated polarized epithelia.

Many ductal epithelia secrete fluid and salts. Determination of ductal fluid modification by secretory epithelia has posed a challenge for researchers for many years (Ishiguro et al. 2009; Bridges 2012; Lee et al. 2012). Ideally, use of dissected ductal tissues would be optimal for study (Ishiguro et al. 1996). However, their preparation is challenging, requires additional specialized equipment, and installation of ductal tissues in an imaging chamber is difficult. There is also limited access to both sides of the epithelial monolayer with electrodes and perfusion of test solutions. Therefore, the present knowledge on ductal ion and fluid secretion has come from single‐cell studies, measurement of monolayer short‐circuit current in Ussing chamber studies, isotope flux, and acid–base titration using the pH‐stat method for proton and bicarbonate secretion (Steward et al. 2005). The latter three methods were designed to use relatively large chamber volumes and commercial insert culture systems. Buffer volume could be considered infinite with regard to the surface area of the epithelium under investigation. In vivo, the volume of fluid in duct systems, aside from the obvious exception of the main ducts, is typically small. Therefore, secreted ions accumulate and modify their gradients. In addition, ephemeral ions such as HCO_3_^−^ and H^+^ change local pH and operation of ion transport mechanisms (Devor et al. 1999). This is especially relevant for research of distal small ducts and airway epithelia, where the small duct lumen and height of the airway surface liquid (approximately 10 *μ*m) is difficult to investigate without perturbation and potential release of autocrine signaling molecules (Grygorczyk and Hanrahan 1997). Our disk culture system is more suitable to measure salt secretion together with these feedback mechanisms. Our disk membrane utilizes a luminal volume of about 3 *μ*L (surface area of 30 mm^2^ × height of 0.09 mm) and has a low ratio of volume to surface area (0.1). In contrast, a typical Ussing chamber setup using a 113 mm^2^ membrane and a luminal chamber volume of 6000 *μ*L has the ratio of about 60, 600 times larger than our system using a disk membrane. This level of dilution makes it difficult to measure secretory products directly.

### Further applications of the disk membrane culture

Our disk membrane culture and chamber systems allow a variety of imaging and electrophysiological experiments. For example, we were able to measure paracellular leakage of fluorescein after ethanol‐induced disruption of PDEC monolayers (Seo et al. 2013). Epithelial monolayers prepared on disk membranes were convenient for confocal inspection (see Fig. 7) and immunocytochemical staining. Unlike commercial filter membrane culture insert systems, which need to be cut from their supports, our disk membrane can be mounted directly onto a glass slide. For cells that do not form differentiated monolayers, the filter membrane can be replaced by a round coverslip for a better optical clearance from both sides.

Based on amperometric experiments using microelectrodes (Fig. 10), we predict that patch‐clamp recording is feasible with single PDEC in a differentiated monolayer (Hamill et al. 1981). Since the plasma membrane of the cell to be patched is not clear as one visualizes through the glass chip, one may need to rely on an increase in electrical resistance for pipette contact on the membrane.

**Figure 11.**
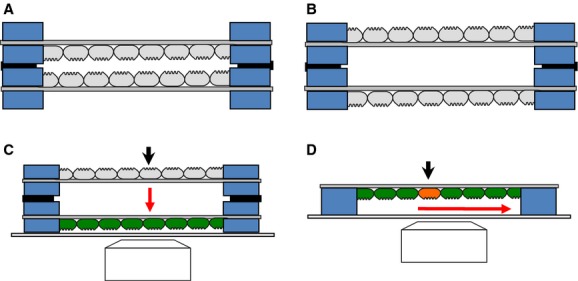
Various applications of epithelial monolayer on disk membrane. Artificial ducts can be constructed in ‘inside‐in’ (A) or ‘inside‐out’ configuration (B) by stacking two disk membranes with a thin sealing grease layer between them (black). (C) A possible experiment using the artificial ducts. Paracrine secretion from the upper monolayer upon stimulation (black arrow) can diffuse through a narrow gap and activate the lower monolayer (green). If the secreted transmitter such as ATP activates intracellular signals such as Ca^2+^, the cells act as a cell sensor. (D) One can monitor intercellular signals such as Ca^2+^ propagating from a cell through the monolayer and Ca^2+^ synchronization between neighboring cells via gap junctions (e.g., Movie S2).

In addition, we envision the possibility that two disks with epithelial monolayers can be stacked in various sandwich orientations (Fig. 11): two luminal sides directed toward each other as found in ductal tissues (‘inside‐in’ configuration, Fig. 11A), or two facing serosal sides (‘inside‐out’ mode, Fig. 11B). These artificial ducts could be used to study different mechanisms of epithelia such as release of autocrine and paracrine transmitters. Since the dilution of secreted molecules is minimal, the transmitters may activate the G‐protein‐coupled receptors coupled to PLC or adenylyl cyclases in the cells on the other side (Fig. 11C) or in the neighboring cells (Fig. 11D). In that case, Ca^2+^ or cAMP detected by imaging techniques will report the autocrine and paracrine release (Jung et al. 2010).

In summary, our disk membrane system using thin O‐rings is optimal platform for imaging and manipulation of live or fixed cells supported by filter membranes.

## Acknowledgments

We thank Drs. Jill Jensen and Martin Kruse for comments on the manuscript and Mr. Eric Martinson for technical assistance.

## Conflict of Interest

Authors have no conflict of interest to declare.
